# No Money No Time Culinary Nutrition Website eHealth Challenge: A Pre-Post Evaluation of Impact on Diet Quality, Food Expenditure, and Engagement

**DOI:** 10.3390/nu16172950

**Published:** 2024-09-02

**Authors:** Rebecca A. Collins, Lee M. Ashton, Tracy L. Burrows, Melinda Hutchesson, Marc T. P. Adam, Erin D. Clarke, Clare E. Collins

**Affiliations:** 1 School of Health Sciences, College of Health Medicine and Wellbeing, The University of Newcastle, Callaghan, NSW 2308, Australia; lee.ashton@newcastle.edu.au (L.M.A.); tracy.burrows@newcastle.edu.au (T.L.B.); melinda.hutchesson@newcastle.edu.au (M.H.); erin.clarke@newcastle.edu.au (E.D.C.); 2 Food and Nutrition Research Group, Hunter Medical Research Institute, New Lambton Heights, NSW 2305, Australia; 3 School of Education, College of Human & Social Futures, The University of Newcastle, Callaghan, NSW 2308, Australia; 4 Active Living Research Program, Hunter Medical Research Institute, New Lambton Heights, NSW 2305, Australia; 5 School of Information and Physical Sciences, College of Engineering, Science and Environment, The University of Newcastle, New Lambton Heights, NSW 2305, Australia; marc.adam@newcastle.edu.au

**Keywords:** nutrition, diet quality, eHealth

## Abstract

No Money No Time (NMNT) is a culinary nutrition website designed to optimize diet quality. The primary aim was to evaluate the impact of an online targeted nutrition challenge email campaign that encouraged engagement with NMNT and goal setting to improve diet quality and weekly food expenditure. A secondary aim was to assess NMNT engagement. Australian adults ≥18 years were recruited to the eHealth nutrition challenge delivered via weekly emails. Diet quality was assessed using the Healthy Eating Quiz (HEQ) diet quality tool. Engagement was assessed using email open and click-through rates. Intention-to-treat (ITT) analysis was conducted using mixed effects linear regression. Of 481 adults (49.7 ± 13.9 years, 84% female) who enrolled 79 (16%) completed the challenge. ITT results indicated statistically significant 6-week increases in diet quality score (+3.8 points *p* ≤ 0.001, *d* = 0.58) with sub-scale improvements in vegetables (+0.9 points, *p* = 0.01, *d* = 0.32), fruit (+1.2 points, *p* ≤ 0.001, *d* = 0.55), and dairy (+0.9 points, *p* ≤ 0.001, *d* = 0.58). There were significant post-challenge reductions in household spending on takeaway/snacks/coffee of AUD 8.9 per week (*p* = 0.01, *d* = 0.29), body weight reduction (−0.6 kg, *p* = 0.03, *d* = 0.26), and BMI (−0.2 kg/m^2^ *p* = 0.02, *d* = 0.28). The email open rate remained constant at around 67% (56% to 75%), with an average click-through rate of 18% (7.1% to 37.9%). The eHealth nutrition challenge significantly improved diet quality while reducing BMI and money spent on discretionary foods. Strategies to scale the challenge should be tested as an innovative population strategy for improving diet quality, health indicators, and managing household food budgets.

## 1. Introduction

Each year, 27,500 Australians die preventable deaths due to unhealthy diets [1], while seven million Australians live with at least one diet-related chronic disease [2]. Primary dietary risks for mortality worldwide are excessive intakes of sodium, red meat, and trans fat, and low intake of wholegrains, legumes, fruit, fiber, nuts, seeds, and vegetables [3]. Therefore, broad-reach strategies to improve dietary patterns and the quality of individuals’ diets are needed to improve the eating habits of both individuals and whole populations. Diet quality refers to the nutritional quality and variety of an individual’s food choices within nutrient-rich core food groups (vegetables, fruits, wholegrains, dairy, lean meats/vegetarian alternatives) that are recommended in national dietary guidelines with diet quality and is an important determinant of overall health, and risk of chronic disease [4,5].

Recent systematic reviews have demonstrated that post-intervention improvements in diet quality are possible and can facilitate improved health outcomes, with higher diet quality associated with more optimal cardiovascular health and reduced cumulative population healthcare costs [6,7,8,9]. A review by Nair et al. (2016) found that improved diet quality as the nutrition intervention strategy is advantageous when working across cultural groups due to promoting a greater variety of nutrient-dense core foods rather than limiting discretionary choices [10]. As well as the physical and mental improvements associated with improved diet quality, studies have shown that greater frequency and variety of vegetable and fruit intake is associated with lower health care costs and number of health care claims over fifteen years in mid-aged women in the Australian Longitudinal Study on Women’s Health [9]. A recent study modeling Australian food systems and current (sub-optimal) Australian dietary intakes projected that 2025 population health costs will exceed three billion Australian dollars [11]. Barriers to improving diet quality include food cost as a major contributor to nutrient-poor food choices [12]. A review by Darmon and Drewnowski (2015) found people on lower incomes choose cheaper, less nutrient dense food, with fewer vegetables and fruit [12]. However, two cross-sectional surveys in over 4300 adults aged 20–65 years in the United States found diet quality in adults could be improved by countering the perception of food cost as a barrier to healthy eating [13].

To date, reviews of eHealth interventions have identified poor evaluation of the impact of intervention on health outcomes [14]. Systematic reviews have identified research gaps in the evaluation of engagement and reach of eHealth interventions and optimal delivery mode [15,16]. Email-delivered dietary interventions are low-cost and -burden interventions, hence, ideal for population groups. However, thus far, they have demonstrated limited improvement in dietary quality and have not specifically evaluated engagement with the email content beyond self-report data [16,17]. Studies including mobile applications have demonstrated diet quality improvements in adults, but more targeted and longer-term eHealth interventions are required [18]. Similarly, a review on the effectiveness of brief nutrition interventions in adults reported that instructional interventions, where participants received direct feedback, were more effective in achieving behavior change compared to interventions that were only educational and/or lacked personalization [19].

A previous evaluation of the diet quality of adolescents and adults who completed the online Healthy Eating Quiz (HEQ), embedded within the No Money No Time (NMNT) website (https://nomoneynotime.com.au, accessed on 4 June 2024), who received brief personalized feedback, demonstrated that diet quality increased over time following receipt of results [20]. This also highlights the potential for population impact if a web-based, publicly available, brief dietary assessment tool and supporting resources were promoted to assist in improving population diet quality, particularly given the current poor dietary intake patterns of Australians [20,21,22]. Therefore, the primary aim of the current study was to evaluate the impact of a targeted 6-week eHealth healthy eating nutrition challenge on diet quality and weekly food expenditure. The secondary aim was to assess engagement with the weekly email campaign content.

## 2. Materials and Methods

### 2.1. Study Design and Participants

This single-arm, pre–post study recruited adults ≥18 years from Australia to participate in a 6-week eHealth brief nutrition challenge delivered via weekly emails that directed participants to a culinary nutrition website, No Money No Time (NMNT) (https://www.nomoneynotime.com.au, accessed on 4 June 2024). Recruitment occurred from 30 August 2023 until 17 September 2023. Individuals who completed the HEQ during this time via the unique nutrition challenge weblink and consented to have their data used for research purposes were automatically signed up for the challenge and included in the analysis. This project was approved by The University of Newcastle Human Research Ethics Committee (H-2018-0512).

The only eligibility criteria were aged ≥ 18 years, residing in Australia, and having completed the baseline HEQ data. All participants were required to set up a profile before completing the HEQ, where they provided consent for using their data for research purposes. Respondents were excluded from the analysis if they did not provide completed baseline HEQ data. Recruitment occurred via social media (Facebook, Instagram, twitter/X) targeted to the general public, and email campaigns to those with a previously registered HEQ profile. Participants were advised in the advertising campaign posts and emails that by completing the HEQ, they were providing consent to participate in the 6-week challenge.

At the start of each challenge week, participants received an email (Figure S1) with links to targeted NMNT content and recipes relevant to the challenge weekly theme (Figure S1), plus access to additional resources to assist them in improving their dietary patterns in a timely and cost-efficient way.

### 2.2. Data Collection Tools

Data collection occurred via an online diet quality tool—the HEQ [23], accessed via an individualized source link specific to the nutrition challenge. NMNT website analytics were collected via Active Campaign software version 3 [24].

#### 2.2.1. The Healthy Eating Quiz

The HEQ is the online version of a validated diet quality index, the Australian Recommended Food Score. The HEQ comprises 70 questions from a previously validated food frequency questionnaire mapped to the Australian Dietary Guidelines food groups [22,25,26]. Diet quality scores from the HEQ are scored from zero to 73 points and can be disaggregated into eight subscales of vegetables (0–21), fruit (0–12), meat (0–7), vegetarian protein foods (0–6), breads and cereals (0–13), dairy foods (0–11), water (0–1), and condiments (0–2) [23,26]. If respondents reported following a vegetarian diet, they were assigned a score of zero for meat questions, with points doubled for vegetarian options selected as consumed “at least once per week or more” and one bonus point awarded if both soybeans, tofu, and other beans, lentils were scored as “at least once per week or more”. The HEQ takes less than 10 min to complete; then, following completion of the questions and automated scoring, a participant obtains their scores with a comparison of data compared to age and gender population norms, with scores categorized into 4 quantiles from lowest to highest as follows: “needs work” (<33), “getting there” (33–38), “excellent” (39–46), or “outstanding” (47+) [22]. Alongside this, there is the provision of a personalized brief nutrition report (Figure S2) with brief advice and NMNT website links provided to participants on ways to improve their diet quality.

#### 2.2.2. The No Money No Time Website

No Money No Time (NMNT) (http://nomoneynotime.com.au, accessed on 4 June 2024) is a purpose-built culinary nutrition website targeting young adults and providing evidence-based nutrition information to users of all age groups. NMNT is freely accessible, and users have access to the HEQ, regularly updated recipes, downloadable healthy eating resources, e-books, and evidence-based short-read articles. The website focuses on countering time and financial limitations experienced by young adults and the wider population to assist them in improving diet quality and overall health, despite budgetary constraints.

### 2.3. Demographics

HEQ demographics collected via fixed response questions included gender, age, vegetarian status (yes or no), household data (number of people and weekly household grocery expenditure, expenditure on food purchased away from home, and weekly per person expenditure on groceries), weight, and height which was then converted to Body Mass Index (BMI). Participant motivation for participating in the nutrition challenge was also collected via the HEQ. Postcode data is collected and matched to the Australian Bureau of Statistics, Index of Relative Socio-economic Advantage and Disadvantage (IRSAD) data [27]. IRSAD quintiles were used for the current study, with one indicating the most disadvantaged and five the most advantaged in terms of economic and social conditions.

### 2.4. Data Analysis

For baseline characteristics, data checking and exploratory data analysis involved the generation of tables and frequency distributions for categorical variables and summary statistics for continuous variables. To investigate the impact of the 6-week nutrition challenge, intention-to-treat mixed-effects linear regression, unadjusted and adjusted for gender, age, ISRAD, previous quiz completion, and vegetarian status, was conducted to assess change in diet quality, food expenditure, and self-reported weight and BMI from baseline to the 6-week follow-up time period. For all models, statistical significance is assessed at the 5% level, and effect sizes are calculated using Cohen’s *d* (*d* = M1-M2/σ pooled). To interpret the effect sizes, the following cutoffs were used: <0.3 small, 0.3 to 0.5 medium, >0.5 large [28]. Missing data was handled using the mixed modeling framework. Internal reliability was analyzed via Cronbach’s alpha values ranging from 0 to 1, with a score closer to 1 indicating higher reliability. All statistical analyses were scripted using STATA v17 (StataCorp LLC, College Station, TX, USA).

## 3. Results

A total of 625 individuals commenced the HEQ and joined the 6-week nutrition challenge during the recruitment period (30 August 2023 until 17 September 2023). Of these, 84.0% (n = 525) completed the HEQ at baseline, with 481 meeting all eligibility criteria to be entered into the 6-week eHealth nutrition challenge (Figure S3). On average, participants were 49.7 ± 13.9 years of age and were living in areas of middle socio-economic status (mean IRSAD: 3.3 ± 1.3). Most were female (84%) and classified as being non-vegetarian (91%). The key motivators for undertaking the healthy eating quiz were “To achieve or maintain a healthy weight” (41.6%), followed by “To feel better or improve wellbeing” (30.8%) and “To know more about how to eat better” (12.9%). The demographic characteristics of study participants are summarised in Table 1.

A total of 16% (n = 79) of participants completed all post-challenge surveys at the end of the 6-week nutrition challenge.

### 3.1. Change in Diet Quality, Food Budget, Weight, and BMI

Frequency and summary tables corresponding to diet quality (mean (SD) 37.7 (9.3)), food expenditure (weekly household spend on groceries: mean (SD) AUD 191.5 (106.0) weekly household spend on takeaway: AUD 65.3 (53.9)), weight (mean (SD) 79.3 kg (19.9)) and BMI (mean (SD) 28.5 kg/m^2^ [7.3]) are stratified by timepoint in Table 2.

Results from the mixed effects linear regression models, with 95% confidence intervals (95% CI), *p*-values, and Cohen’s d effect sizes for both fully adjusted and unadjusted models are presented in Table 3. Involvement in the 6-week challenge demonstrated statistically significant increases in several diet quality outcomes post-intervention. In the unadjusted models total HEQ score increased by a mean of 3.8 (95% CI = 2.4–5.3, *p*-value ≤ 0.001, *d* = 0.58) points over the 6-weeks, compared with baseline with statistically significant improvement in the vegetable sub-scale (+0.9 points, 95% CI = 0.3–1.5, *p*-value = 0.01, *d* = 0.32), fruit sub-scale (+1.2 points, 95% CI = 0.7–1.7, *p*-value ≤ 0.001, *d* = 0.55), and dairy sub-scale (+0.9 point increase, 95% CI = 0.6–1.2, *p*-value ≤ 0.001, *d* = 0.58). These all remained statistically significant in the fully adjusted models. There were no statistically significant changes observed in the HEQ sub-scales for meat or grains sub-scale, while improvement in the vegetarian protein sub-scale was statistically significant in the fully adjusted model only (+0.4 points, 95% CI = 0.0–0.8, *p*-value = 0.04, *d* = 0.32).

For the questions regarding money spent in the weekly food budget post-completion of the 6-week nutrition challenge, there was a statistically significant reduction in weekly household spend on takeaway/snacks/coffee and meals out by AUD 8.9 (95% CI = (−AUD 15.7–2.0), *p*-value = 0.01, *d* = 0.29), which remained significant in the fully adjusted model. No significant changes were observed for the weekly household spend on groceries/at the supermarket or weekly spend on household groceries per person.

Involvement in the 6-week nutrition challenge led to statistically significant decreases in self-report weight, with a mean 0.6 kg reduction in weight (95% CI = −1.1–0.1, *p*-value = 0.03, *d* = 0.26) equating to a BMI reduction of 0.2 kg/m^2^ (95% CI = −0.4–0.0, *p*-value = 0.02, *d* = 0.28). Both remained statistically significant in the fully adjusted models (Table 3).

### 3.2. Engagement with Weekly Emails during the Challenge

The weekly engagement performance data for the challenge emails, as indicated by open rate, click through rate, unsubscribe rate and bounce rate is provided in Table 4. Across the six weeks the email open rate remained relatively constant and high with a drop of 7.9% comparing open rate from week one emails to week six emails. Although click through rates remained high, there was a reduction in click through rate as the weeks progressed, with a 30.8% reduction from week one to week six. Both unsubscribe rates and bounce rate remained relatively low and constant throughout.

## 4. Discussion

The primary aim of the current study was to evaluate the pre–post change in diet quality of adults who enrolled in the NMNT 6-week nutrition challenge. The results demonstrated that this brief email-delivered intervention was effective in improving diet quality, especially regarding the variety of vegetables, fruit, and dairy products, and in reducing household expenditure on takeaway foods, beverages, and meals eaten away from home. Of note, was an unexpected small but statistically significant reduction in mean body weight of 0.6 kg over the 6 weeks. Given that 42% of participants in the eHealth nutrition challenge indicated their main reason for participation was “to achieve or maintain a healthy weight”, it was anticipated that any weight change effect would likely be counterbalanced by goals set by the other 60% of participants. However, the weight change result aligns with a meta-analysis of eHealth interventions that found they can produce significant weight loss, with the eHealth intervention of less than 6 months duration demonstrating significantly greater weight loss than interventions with a duration greater than 6 months [29,30]. Meta-analyses of eHealth interventions indicate that more intensive weight loss interventions (3–12 months) achieve around 0.1 kg weight reduction per week, consistent with the current study, albeit using a much less intensive intervention [29,30].

In the current study, the mean baseline HEQ score was 37.7 points from a maximum of 73 points. This is higher compared to previous studies utilizing the HEQ in large studies of adolescents and adults, which reported mean scores in these samples of 34.1 [22] and 33.9 points, respectively [20]. The higher baseline HEQ scores of the nutrition challenge participants could be due to attracting participants who have a greater interest in nutrition or those who have already engaged with the NMNT website (54.3%). Therefore, participants potentially had previously made dietary changes leading to higher diet quality, particularly as HEQ completion triggers receipt of personalized, brief advice on how to improve diet quality, along with links provided to NMNT resources and information, which have been previously shown to improve diet quality [20]. Another consideration is the higher percentage of female participants (84.2%) compared to males (15.4%). A recent analysis of HEQ data in young Australian adults by Fenton et al. (2024) showed that diet quality in females was significantly higher than in males (33.1 ± 8.6 vs. 31.4 ± 9.3 points out of 73; *p* < 0.001) [31].

Given the large proportion who did not repeat the final survey, intention-to-treat (ITT) principles could have been expected to mean there was no statistically significant change in scores over time. However, using ITT, the challenge participants achieved significant increases in their HEQ scores at the 6-week follow-up, and those who had previously engaged with the NMNT website were able to further improve diet quality. Additionally, the changes in total HEQ scores are higher (3.8 points versus 2.3 points) than those reported previously among participants who had repeated the HEQ over time previously [20]. The multiple significant improvements for participants in the eHealth challenge include an average total HEQ score increase of 3.5 points, reduced spend on takeaway food of around AUD 8 per week, and reduction in body weight of 0.6 kg, indicates the effectiveness of a brief eHealth nutrition interventions which could potentially contribute to a reduction in chronic disease risk in adults if administered at scale.

The nutrition challenge also focused on food expenditure, with three of the weekly emails including tips on food budgeting and achieving savings on weekly food and grocery spending, especially food and beverages consumed away from home. The current cost of living pressures, defined as a decrease in disposable income across the population in multiple countries [32], means that saving money is a high priority for many people, and this can be achieved by rationalizing money allocated to the household food budget. The challenge results demonstrated that the current 6-week email campaign led to reduced money spent by participants on food eaten away from home, with the fully adjusted model showing participants spending on average AUD 8 less per week on takeaway, snacks, coffees, or meals out. The lack of significant changes in the weekly household or per person spend on groceries suggests this was achieved without a change in grocery spend. This indicates the potential for less household food waste, as more people may prepare pre-bought food at home or reduce overall food intake [33]. Additionally, this is likely to have contributed to the average 0.6 kg weight reduction reported by participants, as has been demonstrated in a previous eHealth weight loss randomized controlled trial where higher diet quality was associated with higher rates of weight loss [34]. However, It should be noted that all weight measures collected were self-reported by participants. It is well documented that both men and women tend to underestimate their weight when self-reporting, with women often underreporting their weight by −0.1–−6.5 kg [35]. Takeaway food and meals purchased away from the home are more likely to have a lower vegetable content [36], and fruit is not a commonly purchased snack away from the home. As takeaway food is often high in other ingredients, including sodium, saturated fats, and trans fats [37], the reduction in the purchasing of takeaway foods achieved by the participants indicates that online challenges can be an effective tool in changing dietary habits that contribute to an increased risk of cardiovascular disease, particularly when the reduction in body weight is also considered.

The results of the 6-week challenge demonstrate the effectiveness of the challenge’s email marketing campaign and highlight the ability of eHealth challenges to reach low socio-economic populations. This is important to note, as lifestyle change programs are often inaccessible to harder-to-reach populations, increasing health inequities [38]. The challenge consistently demonstrated email open rates and email click-through rates well above the worldwide average of email marketing campaigns [39]. The average email open rate for the NMNT 6-week nutrition challenge was 66.8%, which far exceeds the average open rate for all online sectors in 2022 (21.5%) and especially when compared to the very low open rates of the individual sectors of healthcare services (3.0%) and wellness/fitness (1.2%) [39]. Similarly, exceptional results were achieved regarding email click-through rates, with an average rate of 18.1% in the challenge compared to average all sector rates of 10.5%, health care services (3.0%), and wellness and fitness (1.2%) [39].

The challenge retention rate was 16%, which is within the range (16–98%) of retention reported in a meta-analysis of eHealth interventions, although at the lower end [30]. The nutrition challenge results are substantial given that it was highly self-directed, with participant contact only made once per week via a challenge email. Previous research suggests that self-management in eHealth weight-based interventions is possible; however, participant ability to implement health change by self-direction is greatly influenced by participants’ level of self-efficacy (e.g., social support and patient-centered interactions) [40]. The current nutrition challenge focused on supporting participants in engaging in personalized goal setting linked to the HEQ and facilitating increased self-efficacy. Future eHealth nutrition interventions should consider including content to support participants in increasing their social support systems and participant-centred interactions (e.g., facilitated personal forums). The current study did not include financial incentives for participation, given the challenge included a focus on food expenditure and budgeting future challenges could also consider whether offering financial incentives for participation and evaluate whether it impacts retention.

The strengths of the current study include the fact that the eHealth nutrition challenge was free and easily accessible and linked to the No Money No Time website. In addition, participants were able to instantly access a personalized nutrition report once the HEQ was completed, at baseline and follow-up, and at their own convenience, which minimized both researcher and participant cost and burden. The nutrition challenge was completed in September and October 2023, which is spring in Australia, and people may potentially feel more motivated to engage in lifestyle changes [41]. Limitations were that all data collected was self-reported and that the majority of participants were female, meaning the interpretation of results for males and non-binary individuals should be considered carefully. The HEQ does not collect data on participants’ ethnicity or cultural backgrounds, which may also impact the interpretation of the results. The assumptions underlying the linear mixed models were not analyzed, and, therefore, results should be interpreted cautiously from this perspective. Additionally, the pre–post design, albeit real-world, did not include comparison with a control group.

## 5. Conclusions

The NMNT eHealth nutrition challenge represents an easy-to-implement, low-cost, innovative intervention for improving diet quality while reducing discretionary spending on food and body weight among Australian adults. The current study demonstrates the ability to support people in making healthy lifestyle changes with a low burden of commitment. Given the low cost and low burden of the nutrition challenge, ease of access to the HEQ tool and the online resources accessible via NMNT can be effectively used in clinical settings and promoted at levels to benefit public health to improve diet quality in individuals and populations. The unexpected finding for weight reduction demonstrates that promoting enhanced diet quality by targeting individual motivators for improving nutrition may have additional benefits. Further research should evaluate the change in food/cooking patterns and household waste, as this may have occurred in parallel with the reduction in spending on food away from the home. The effectiveness of the challenge should be assessed in larger populations, along with incentivization strategies to promote completion of the post-challenge evaluation survey. Feedback from end-users will also be incorporated into future versions of the eHealth challenge to ensure the promotion of healthy equity, and strategies will be implemented to ensure the inclusion of those with reduced access to online services.

## Figures and Tables

**Table 1:** Demographics of adults enrolled in the NMNT 6-week eHealth nutrition challenge (n = 481).

Variable	Category	Total Sample
	n	481
Gender (n%)	Male	74 (15.4%)
	Female	405 (84.2%)
	Another gender identity	2 (0.2%)
Age (years)	Mean (SD *)	49.7 (13.9)
	Median (Q1, Q3 **)	50.6 (38.7, 60.8)
Vegetarian n (%)	Yes	43 (8.9%)
	No	438 (91.0%)
Number of people’s main meals shared with n (%)	Only themselves	126 (26.4%)
	With one other person	184 (38.6%)
	With 2+ other people	167 (35.0%)
First time taking the healthy eating quiz n (%)	Yes	220 (45.7%)
	No	261 (54.3%)
What is your main reason for taking the healthy eating quiz? n (%)	To know more about how to eat better	62 (12.9%)
	To find out whether my diet is healthy	40 (8.3%)
	To achieve or maintain a healthy weight	200 (41.6%)
	To perform better in sport	10 (2.1%)
	To feel better or improve wellbeing	148 (30.8%)
	Other	21 (4.4%)
Number of people living in household n (%)	1	122 (25.4%)
	2	168 (34.9%)
	3	61 (12.7%)
	4	90 (18.7%)
	5+	40 (8.3%)
Index of relative socio-economic advantage and disadvantage (quintile) n (%)	1 (most disadvantaged)	50 (10.5%)
	2	87 (18.3%)
	3	110 (23.2%)
	4	115 (24.2%)
	5 (most advantaged)	113 (23.8%)
	Mean (SD)	3.3 (1.3%)

* SD—Standard Deviation ** Q—Quartiles.

**Table 2:** Median (Q1, Q3) pre–post 6-week eHealth nutrition challenge data for adults participating in the No Money No Time (NMNT) email campaign (n = 481) for Healthy Eating Quiz diet quality scores, food expenditure, and self-reported height, weight, and BMI. Data is median (Q1, Q3).

		Time Point		
Variable	Category	Baseline (n = 481)	Post-Challenge: 6-Weeks (n = 79)	Internal Reliability
Total ARFS score */73	median (Q1, Q3 **)	38 (32, 44)	42 (39, 48)	0.6
Vegetable sub-scale/21	median (Q1, Q3)	14 (12, 17)	16 (14, 18)	0.6
Fruit sub-scale/12	median (Q1, Q3)	6 (4, 7)	7 (5, 9)	0.7
Meat/flesh sub-scale/7	median (Q1, Q3)	3 (2, 4)	4 (3, 5)	0.7
Plant-based protein sub-scale/6	median (Q1, Q3)	3 (2, 4)	4 (2, 4)	0.7
Grains sub-scale/13	median (Q1, Q3)	6 (5, 8)	6 (5, 8)	0.7
Dairy sub-scale/11	median (Q1, Q3)	4 (2, 5)	5 (3, 6)	0.7
Weekly household spend on groceries/at the supermarket (AUD)	n	477	78	
	Mean	AUD 191.5 (106.0)	AUD 174.4 (107.6)	
	Median (Q1, Q3)	180 (100, 250)	150 (100, 200)	
Weekly household spend on takeaway/snacks/coffee and meals out? (AUD)	n	475	78	
	Mean	AUD 65.3 (53.9)	AUD 45.3 (48.0)	
	Median (Q1, Q3)	50 (25, 100)	30 (15, 50)	
Weekly spend on groceries/at the supermarket per person in the household (AUD)	n	477	78	
	Mean	AUD 83.5 (44.9)	AUD 88.6 (40.9)	
	Median (Q1, Q3)	75 (60, 100)	77.5 (62.5, 100)	
Self-reported weight (kg)	n	449	70	
	Mean	79.3 (19.9)	78.1 (18.2)	
	Median (Q1, Q3)	76 (65, 89)	77.5 (66, 86)	
BMI *** (kg/m^2^)	n	445	68	
	Mean	28.5 (7.3)	28.4 (6.0)	
	Median (Q1, Q3)	27.1 (23.9, 31.3)	27.8 (23.8, 31.6)	
BMI category	Underweight	5 (1.1%)	4 (5.9%)	
	Healthy weight	147 (33.0%)	16 (23.5%)	
	Overweight	152 (34.2%)	22 (32.4%)	
	Obese	141 (31.7%)	26 (38.2%)	

* A higher score indicates higher overall diet quality. ** Q—quartiles. *** BMI—Body Mass Index

**Table 3:** Adjusted and unadjusted linear mixed model estimates with 95% CI and *p*-value for change in outcomes.

Outcome	6-Week Change from Baseline (n = 481)			
	Unadjusted Model		Adjusted * Model	
	Estimate (95% CI)	*p*-Value (Cohen’s d)	Estimate (95% CI)	*p*-Value (Cohen’s d)
**Australian Recommended Food Score**				
Total ARFS score/73	3.8 (2.4, 5.3)	**<0.001 (0.58)**	3.5 (2.0, 4.9)	**<0.001 (0.52)**
Vegetable sub-scale/21	0.9 (0.3, 1.5)	**0.01 (0.32)**	0.7 (0.1,1.4)	**0.02 (0.26)**
Fruit sub-scale/12	1.2 (0.7, 1.7)	**<0.001 (0.55)**	1.1 (0.6, 1.6)	**<0.001 (0.48)**
Meat/flesh sub-scale/7	0.1 (−0.2, 0.4)	0.46 (0.08)	0.1 (−0.2, 0.4)	0.57 (0.06)
Vegetarian protein sub-scale/6	0.4 (−0.0, 0.7)	0.06 (0.22)	0.4 (0.0, 0.8)	0.04 (0.23)
Grains sub-scale/13	0.3 (−0.1, 0.7)	0.19 (0.15)	0.3 (−0.1, 0.8)	0.10 (0.19)
Dairy sub-scale/11	0.9 (0.6, 1.2)	**<0.001 (0.58)**	0.8 (0.4, 1.1)	**<0.001 (0.51)**
*Food budget*				
Weekly household spend on groceries/at the supermarket (AUD)	1.2 (−10.6, 13.0)	0.85 (0.02)	2.5 (−9.4, 14.5)	0.68 (0.05)
Weekly household spend on takeaway/snacks/coffee and meals out? (AUD)	−8.9 (−15.7, −2.0)	**0.01 (0.29)**	−7.8 (−14.7, −0.8)	**0.03 (0.25)**
Weekly spend on groceries/at the supermarket per person in the household (AUD)	5.8 (−1.6, 13.3)	0.13 (0.17)	6.1 (−1.4, 13.7)	0.11 (0.18)
*Adiposity*				
Self-reported weight (kg)	−0.6 (−1.1, −0.1)	**0.03 (0.26)**	−0.6 (−1.1, −0.1)	**0.03 (0.26)**
BMI ** (kg/m^2^)	−0.2 (−0.4, −0.0)	**0.02 (0.28)**	−0.2 (−0.4, −0.0)	**0.02 (0.28)**

* Adjusted for gender, age, SES, previous quiz completion and vegetarian status ** BMI – Body Mass Index. Bolded values are those that reached statistical significance.

**Table 4:** Engagement performance of adults participating in the No Money No Time (NMNT) 6-week eHealth nutrition challenge campaign emails.

Email	Open Rate	Click through Rate	Unsubscribe Rate	Bounce Rate
Week 1	75.5%	37.9%	0.6%	0.2%
Week 2	66.9%	23.0%	0.7%	2.4%
Week 3	67.2%	24.6%	0.6%	0.2%
Week 4	66.1%	11.5%	0.6%	0.4%
Week 5	56.4%	11.6%	0.2%	0.2%
Week 6 (email 1)	67.8%	11.1%	0.6%	0%
Week 6 (email 2)	67.6%	7.1%	0.4%	0%

## Data Availability

The raw data supporting the conclusions of this article will be made available by the authors upon request.

## Supplementary Materials

The following supporting information can be downloaded at: https://www.mdpi.com/article/10.3390/nu16172950/s1, Figure S1: Example portion of the email sent to the No Money No Time (NMNT) 6-week nutrition challenge participants in Week 1 of the challenge; Figure S2: Example portion of the personalized brief nutrition report provided to participants upon completion of the Healthy Eating Quiz (HEQ); Figure S3: Participant flow through the No Money No Time (NMNT) 6-week eHealth nutrition challenge email campaign; Table S1: No Money No Time (NMNT) 6-week eHealth nutrition challenge weekly email campaign themes used to guide online content; Table S2: Completers analysis (n = 79) from the No Money No Time 6-week nutrition eHealth challenge. Linear mixed model estimates with 95% CI and *p*-value for change in outcomes.
