# Supplementary material for: No Money No Time Culinary Nutrition Website eHealth Challenge: A Pre-Post Evaluation of Impact on Diet Quality, Food Expenditure, and Engagement
Source: Nutrients. 2024 Sep 2;16(17):2950. doi: 10.3390/nu16172950 (PMC11397432; doi:10.3390/nu16172950)
Supplement: Supplementary file 1 [file nutrients-16-02950-s001.zip › nutrients-3157030-supplementary.pdf]

Table S1: No Money No Time (NMNT) 6-week eHealth nutrition challenge weekly email campaign themes used to guide online content.

Challenge Week	Theme
**Week 1**	Goal setting
**Week 2**	Self-monitoring
**Week 3**	How to increase fruit and vegetable intake
**Week 4**	Self-check in
**Week 5**	Maintaining healthy habits
**Week 6**	Reflection

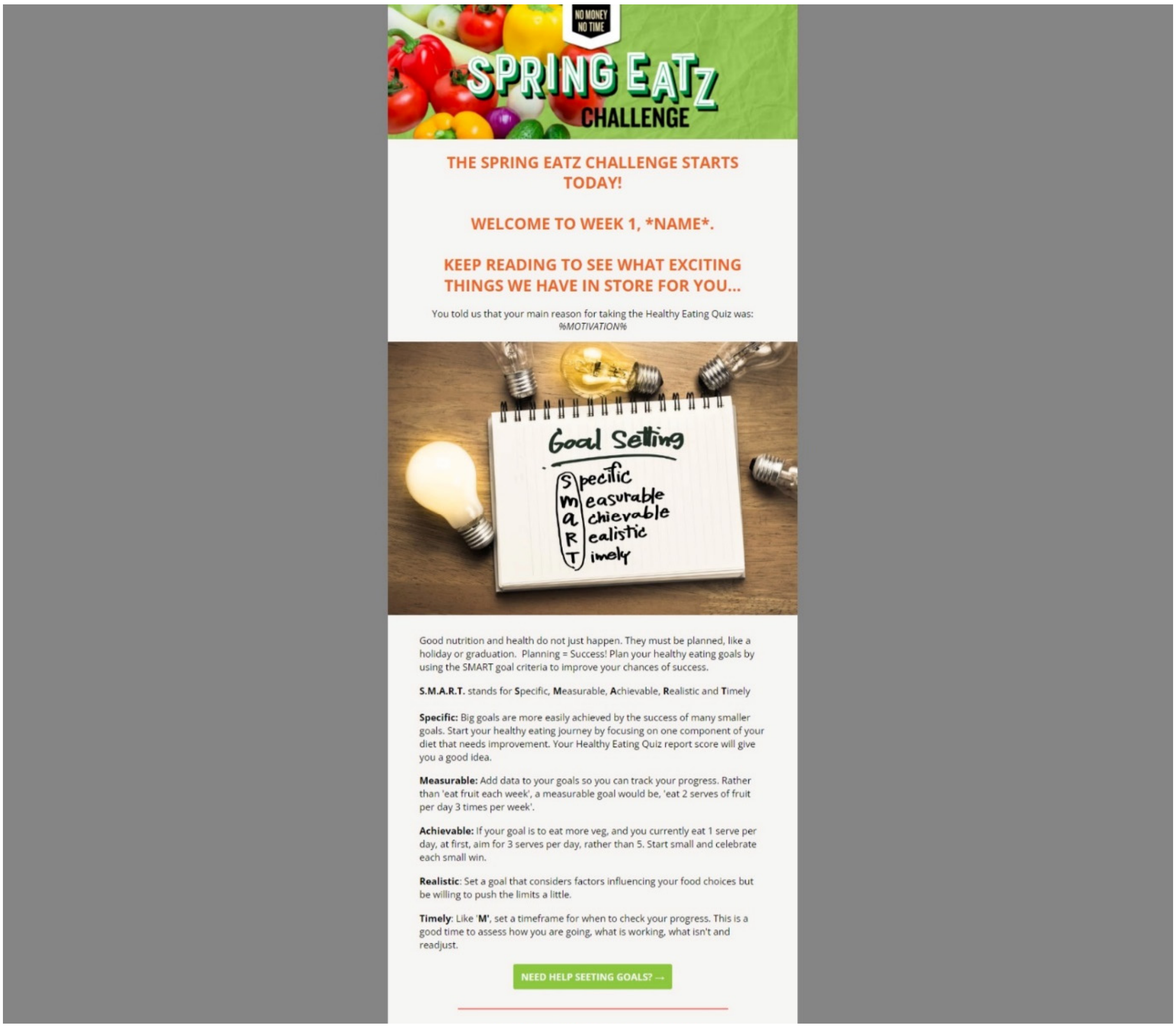

Figure S1: Example portion of the email sent to the No Money No Time (NMNT) 6-week nutrition challenge participants in Week 1 of the challenge

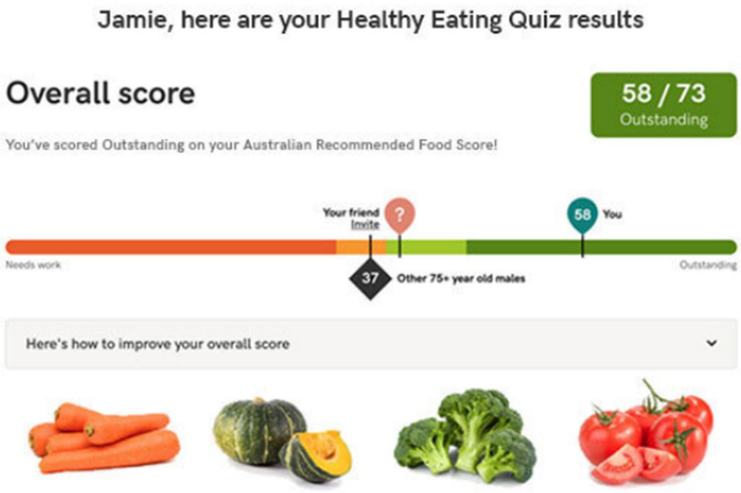

Figure S2: Example portion of the personalised brief nutrition report provided to participants upon completion of the Healthy Eating Quiz (HEQ)

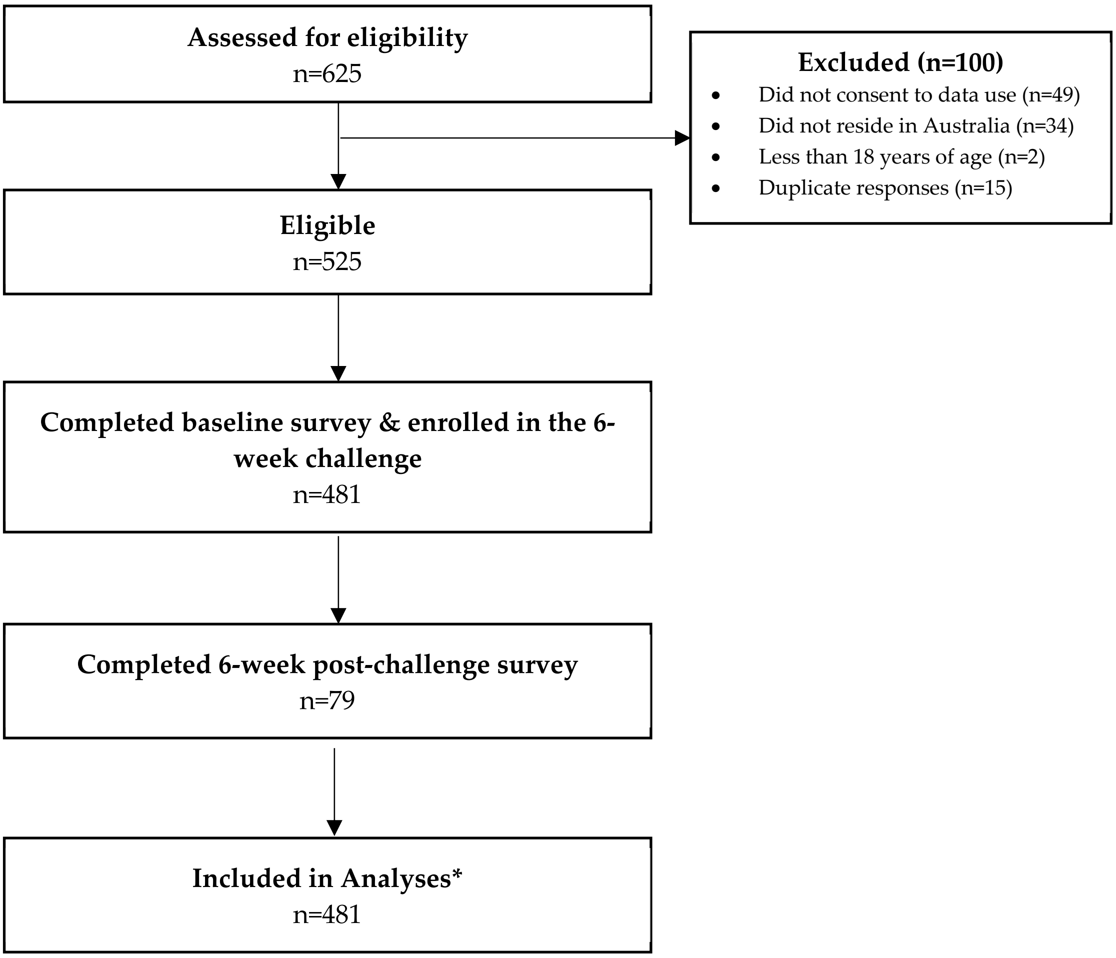

Figure S3: Participant flow through the No Money No Time (NMNT) 6-week eHealth nutrition challenge email campaign. * Missing data was handled using the mixed modelling framework.

Table S2: Completers analysis (n=79) from the No Money No Time 6-week nutrition eHealth challenge. Linear mixed models estimates with 95% CI and p-value for change in outcomes.

	6-week change from baseline (n=79)	
**Outcome**	**Estimate (95%CI)**	**p-value**
Total ARFS score/73	3.1 (1.6, 4.7)	**<0.001**
Vegetable sub-scale/21	0.6 (-0.4, 1.3)	0.07
Fruit sub-scale/12	0.9 (0.4, 1.5)	**<0.001**
Meat/ flesh sub-scale/7	-0.1 (-0.4, 0.3)	0.78
Vegetarian protein sub-scale/6	0.3 (-0.1, 0.7)	0.17
Grains sub-scale/13	0.2 (-0.2, 0.7)	0.36
Dairy sub-scale/11	0.9 (0.5, 1.3)	**<0.001**
Food budget		
Weekly household spend on groceries/at the supermarket (AUD)	3.8 (-8.4, 15.9)	0.54
Weekly household spend on takeaway/snacks/coffee and meals out? (AUD)	-6.5 (-13.4, -0.6)	0.07
Weekly spend on groceries/at the supermarket per person in the household (AUD)	6.1 (-1.7, 13.9)	0.13
Adiposity		
Self-reported weight (kg)	-0.6 (-1.1, -0.6)	**0.03**
BMI (Kg/m^2)	-0.2 (-0.4, -0.3)	**0.02**

Bolded values are those that reached statistical significance.
